# Supplementary material for: The Construction and Exploration of a Comprehensive MicroRNA Centered Regulatory Network in Foxtail Millet (Setaria italica L.)
Source: Front Plant Sci. 2022 May 6;13:848474. doi: 10.3389/fpls.2022.848474 (PMC9121102; doi:10.3389/fpls.2022.848474)
Supplement: Supplementary file 4 [file Data_Sheet_4.zip › Sit-miR5568_Seita.2G376100.1_355_TPlot.pdf]

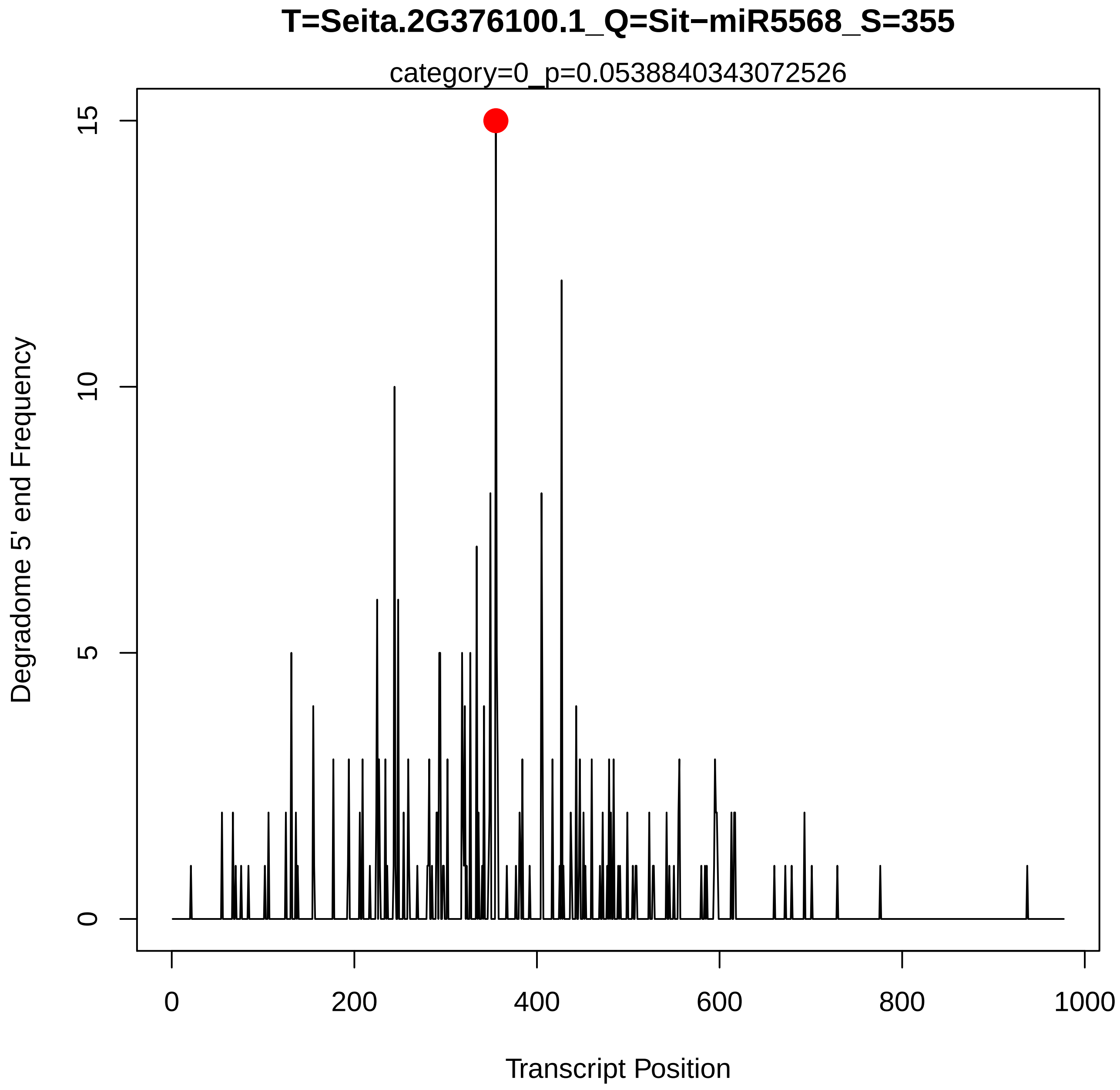

T=Seita.2G376100.1_Q=Sit−miR5568_S=355
category=0_p=0.0538840343072526
Degradome 5' end Frequency
Transcript Position
0
5
10
15
0
200
400
600
800
1000
